# Stage IA undifferentiated uterine sarcoma with SMARCA4 and SMARCA2 co-deficiency: a case report

**DOI:** 10.3389/fonc.2026.1939651

**Published:** 2026-10-02

**Authors:** Rong Ji, Xialin Chen, Juan Lang, Zhongkui Xiong

**Affiliations:** 1 Department of Radiation Oncology, Shaoxing Second Hospital, Shaoxing, Zhejiang, China; 2 School of Medicine, Shaoxing University, Shaoxing, Zhejiang, China; 3 Department of Oncology, Shaoxing People’s Hospital, Shaoxing, Zhejiang, China; 4 Department of Pathology, Shaoxing People’s Hospital, Shaoxing, Zhejiang, China

**Keywords:** co-deficiency, disease-free survival, SMARCA2, SMARCA4, undifferentiated uterine sarcoma

## Abstract

Uterine sarcomas represent a rare and heterogeneous group of malignancies whose management remains clinically challenging. They account for approximately 3%–5% of all uterine malignancies. Undifferentiated uterine sarcoma (UUS) is a subtype of uterine sarcomas. *SMARCA4* is a tumor suppressor gene that is aberrantly altered in approximately 5%–7% of human malignancies. *SMARCA4*-deficient UUS is a highly aggressive, undifferentiated mesenchymal neoplasm associated with a median overall survival (OS) of 7 months. During tumorigenesis, loss of function of either *SMARCA4* or *SMARCA2* is frequently observed; however, concurrent loss of both genes has been reported to be synthetically lethal and is therefore considered unexpected. In this case report, a 52-year-old Chinese woman with early-stage UUS and *SMARCA4* and *SMARCA2* co-deficiency underwent surgical resection followed by adjuvant chemotherapy and achieved a disease-free survival (DFS) of 32 months. This case suggests that patients with UUS harboring *SMARCA4* and *SMARCA2* co-deficiency may derive a survival benefit from early diagnosis and surgical resection. However, owing to the extreme rarity of UUS with co-deficiency of *SMARCA4* and *SMARCA2*, conducting a high-level phase III randomized controlled trial to validate this observation remains impractical.

## 1 Introduction

Uterine sarcomas represent a rare and heterogeneous group of malignancies whose management remains clinically challenging (1). They account for approximately 3%–5% of all uterine malignancies (2). Recently, the World Health Organization (WHO) classified endometrial stromal tumors (ESTs) into four distinct categories: endometrial stromal nodule (ESN), low-grade endometrial stromal sarcoma (LG-ESS), high-grade endometrial stromal sarcoma (HG-ESS), and undifferentiated uterine sarcoma (UUS) (3). Surgical resection remains the cornerstone of treatment for early-stage endometrial stromal sarcoma (4). Due to the rarity of uterine sarcomas, their complex biological behavior, and the limited understanding of their etiology and associated risk factors, the role of adjuvant therapy remains uncertain (5).

The SWI/SNF (SWItch/sucrose non-fermentable) complex is a chromatin-remodeling complex that is frequently mutated in human cancer (6). Loss of one or more *SWI/SNF* complex subunits, including *AT-rich interactive domain-containing protein 1A (ARID1A), ARID1B, SMARCA4 (SWI/SNF-related, matrix-associated, actin-dependent regulator of chromatin, subfamily A, member 4)*, and *SMARCA2*, was identified in 66% (55 of 83) cases of dedifferentiated and undifferentiated endometrial carcinomas (7). Inactivation of the SWI/SNF complex in the endometrial epithelium compromises epithelial integrity (8). The *SMARCA* proteins (such as *SMARCA4* and *SMARCA2*) belongs to the *SWI/SNF* family of evolutionarily conserved ATP-dependent chromatin remodeling complexes that regulate gene expression, DNA repair, and genome stability (9).

SMARCA4-deficient UUS is a highly aggressive, undifferentiated mesenchymal neoplasm associated with a median overall survival (OS) of 7 months (10). It is associated with a poor prognosis and lacks standardized, evidence-based treatment strategies (11). *SMARCA4* and *SMARCA2* co-deficiency defines a distinct subset of rare, aggressive, undifferentiated malignancies, associated with exceptionally poor clinical outcomes (12, 13). Herein, we report the case of a 52-year-old Chinese woman with UUS harboring SMARCA4 and *SMARCA2* co-deficiency who achieved a disease-free survival (DFS) of 32 months.

## 2 Case presentation

On April 30, 2022, a 52-year-old Chinese woman was admitted to the Affiliated Hospital of Shaoxing University with a 3-day history of left lower abdominal pain. Physical examination revealed localized deep tenderness in the left inguinal region. Transvaginal ultrasonography performed on the day of admission demonstrated uterine enlargement measuring 69 × 47 × 58 mm. The myometrium exhibited focal thickening with heterogeneous echotexture. Notably, anterior and posterior myometrial walls were diffusely thickened, and a hypoechoic lesion measuring 20 × 18 mm with ill-defined margins was identified in the posterior wall (**Figure 1A**). Abdominal computed tomography (CT), also performed on April 30, 2022, demonstrated uterine enlargement (**Figure 1B**) and irregular soft-tissue masses within the uterine cavity. On July 18, 2022, contrast-enhanced pelvic magnetic resonance imaging (MRI) revealed localized thickening of the left lateral uterine wall adjacent to the fundal myometrium. An intracavitary lesion measuring 23 × 23 × 19 mm exhibited heterogeneous signal intensity. The interface between the lesion and the anterior myometrium was indistinct, and the lesion involved less than 50% of the myometrial thickness (**Figures 1C, D**). After contraindications had been excluded, the patient underwent hysteroscopic adhesiolysis under general anesthesia on July 18, 2022. Subsequently, on July 23, 2022, she underwent a comprehensive laparoscopic procedure under general anesthesia, including pelvic lymph node dissection, extraperitoneal hysterectomy, bilateral adnexectomy, and pelvic adhesiolysis. The postoperative pathological findings are summarized in **Table 1**. The patient’s paternal family history included a documented case of fatal lung cancer.

**Figure 1:**
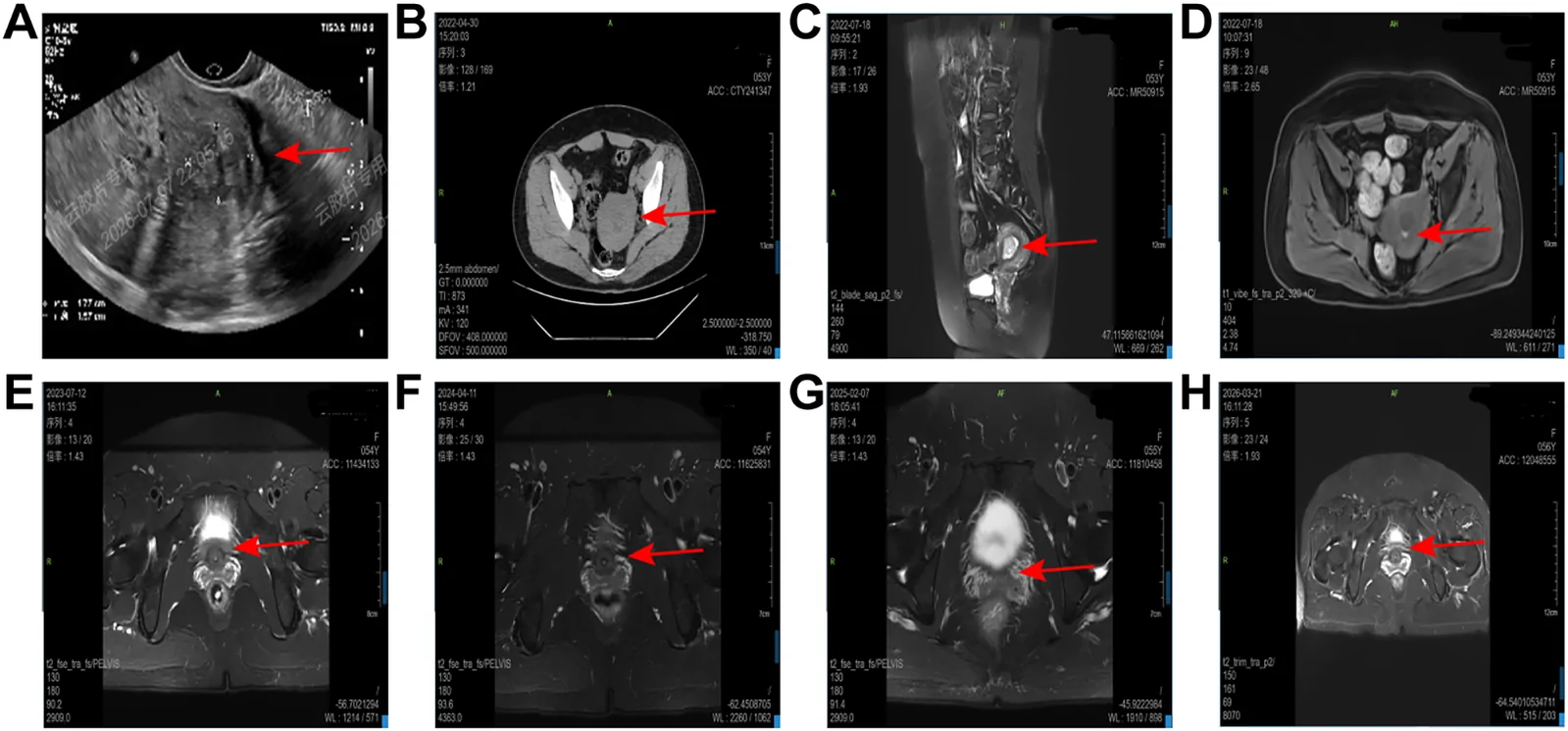
Radiographic findings of the patient. Preoperative imaging of the patient **(A–D)**. Transvaginal ultrasonography performed on April 30, 2022, revealed a hypoechoic lesion measuring 20 × 18 mm in the posterior uterine wall with ill-defined margins **(A)**. Abdominal CT performed on April 30, 2022, revealed uterine enlargement **(B)**. Pelvic MRI performed on July 18, 2022, identified a 23 × 23 × 19 mm intracavitary lesion with heterogeneous signal intensity **(C)**, heterogeneous contrast enhancement, and an ill-defined interface with the anterior myometrium **(D)**. Follow-up pelvic MRI performed between July 21, 2023, and March 21, 2026, demonstrated no evidence of tumor recurrence after adjuvant chemotherapy, indicating continued disease-free status **(E–H)**. MRI performed on July 21, 2023 **(E)**; April 11, 2024 **(F)**; February 7, 2025 **(G)**; March 21, 2026 **(H)**.

**Table 1:** Postoperative pathology.

Date	Postoperative pathology	Immunohistochemical results
July 2022	Postoperative pathology (Intrauterine material): All fragmented tissues were sampled and sectioned. Microscopically, two small pieces of tissue were identified as endometrial polyps, one of which had relatively rich glands and active growth; the remaining tissues were rich in tumor cells, with obvious atypia, frequent mitotic figures, and a few glands with glandular epithelial lining. Based on the immunohistochemical results, the primary consideration was uterine ESS.	ER (3+, 20%), PR (-), Ki67 (hotspot: 60%), CD10 (partly +), Desmin (partly +), CyclinD1 (partly +), Pax-8 (glandular epithelium +), CK7 (glandular epithelium +), SMA (-). ‘Ascites’: Scattered mesothelial cells and inflammatory cells were seen, with no obvious malignant tumor cells.
July 2022 (5 days subsequent to the preceding date)	Postoperative pathology: 1. High-grade uterine ESS with a protruding type on the left uterine wall (tumor size approximately 3.6 × 2.8 × 1.0 cm), with shallow myometrial invasion (< 1/2 myometrium), tumor thrombus in blood vessels, no tumor involvement in the left and right parametrium, and cervical stump. 2. No tumor metastasis in the pelvic lymph nodes (0/13) (left pelvic 0/9, right pelvic 0/4). 3. Adenomyosis. 4. Endometrial polyps. 5. Chronic cervicitis with leiomyoma. 6. Bilateral fallopian tube tissue with mesenteric paramesonephric duct cysts, left ovary simple cyst, right ovary normal.	C: ER (-), PR (-), Ki67 (40%+), CD10 (-), Desmin (focal, +), CyclinD1 (focal, +), CK (pan) (-), D2-40 (blood vessels +), ERG (blood vessels +), CD34 (blood vessels +), CK7 (-), HMB-45 (-), Pax-8 (stroma +). K: Desmin (+), CD10 (k+), S-100 (-), Ki67 (< 1%+), ER (3+, 90%), CD34 (-). Diagnosis: 1. High-grade uterine ESS, IA stage.
September 2022	The pathology consultation opinion: (Uterus) Malignant tumor. Based on morphology (**Figure 2A**) and immunohistochemical results, it was considered an undifferentiated tumor with SMARCA4 expression loss, tending to be SMARCA4 expression loss undifferentiated sarcoma with tumor invasion of the uterine myometrium < 1/2. Vascular invasion (+). No cancer involvement in bilateral adnexa, no cancer metastasis in pelvic lymph nodes (0/13). The endometrium revealed atrophic morphology. Chronic cervicitis with leiomyoma.	AE1/AE3 (-), CK18 (-), ER (+), PR (weak +), GPAX8 (-), SMARCA2 (BRM) (-) (**Figure 2B**), SMARCA4 (BRG1) (-) (**Figure 2C**), SMARCB1 (INI-1) (+) (**Figure 2D**), WTI (-), PMS2 (+), MLH1 (+), MSH2 (+), MSH6 (+), PTEN (weak -), PAX2 (weak -), PD-L1 (-) (**Figure 2E**).

On September 19, 2022, the patient was referred to Fudan University Shanghai Cancer Center for further diagnostic evaluation and management. The postoperative pathological findings are summarized in **Table 1** and **Figure 2**. The postoperative diagnosis was UUS with *SMARCA4* and *SMARCA2* co-deficiency, classified as pT1N0M0 according to the American Joint Committee on Cancer (AJCC)/Union for International Cancer Control (UICC) TNM staging system, 8^th^ edition, corresponding to Stage IA. Adjuvant chemotherapy consisting of intravenous albumin-bound paclitaxel (0.2 g) and gemcitabine (1.6 g) was administered on Day 1 of each 3-week cycle for 4 cycles between September 27 and December 13, 2022. During treatment, laboratory investigations demonstrated hepatic dysfunction, and hepatoprotective therapy was initiated (**Figures 3A, B**). According to the Common Terminology Criteria for Adverse Events (CTCAE) Version 6.0, treatment-related adverse events included Grade 1 alanine aminotransferase (ALT) elevation and Grade 1 aspartate aminotransferase (AST) elevation. The diagnosis and therapeutic timeline for patients is presented in **Figure 3C**. Previously published similar cases are summarized in **Supplementary Table S1**.

**Figure 2:**
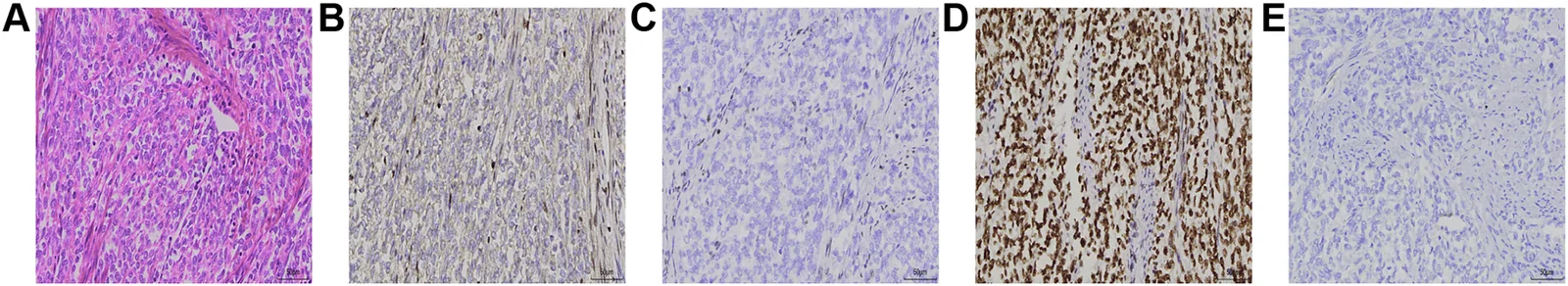
Pathological examination findings, acquired at 400× magnification with a 50-μm scale bar. On the hematoxylin and eosin (H&E)-stained image **(A)**, microscopic examination revealed a tumor with poorly demarcated margins and infiltrative growth into the myometrial wall. The neoplastic cells formed diffuse, densely packed sheets composed of plump, spindle-shaped cells. Notable histopathologic features included marked nuclear atypia, frequent mitotic figures, including atypical mitoses, focal rhabdoid morphology, and intravascular tumor emboli; SMARCA2 (BRM) (-) **(B)**; SMARCA4 (BRG1) (-) **(C)**; SMARCB1 (INI-1) (-) **(D)** and programmed death-ligand (PD-L1) (-) **(E)**. Postoperative histopathological evaluation performed on September 19, 2022, confirmed the diagnosis of UUS with concurrent SMARCA4 and SMARCA2 co-deficiency. The PD-L1 monoclonal antibody clone SP263 was employed for immunohistochemical detection. The INI-1 monoclonal antibody (Cat. No. 0TIR4G9) was purchased from Jiangsu Lanou Medical Co., Ltd., China. The SMARCA2 monoclonal antibody (Cat. No. XSM-455) was obtained from Shanghai Zhongke New Life Biotechnology Co., Ltd., China. The SMARCA4 monoclonal antibody (Cat. No. 121910c81) was purchased from Calt (Jiangsu) Biopharmaceutical Co., Ltd., China.

**Figure 3:**
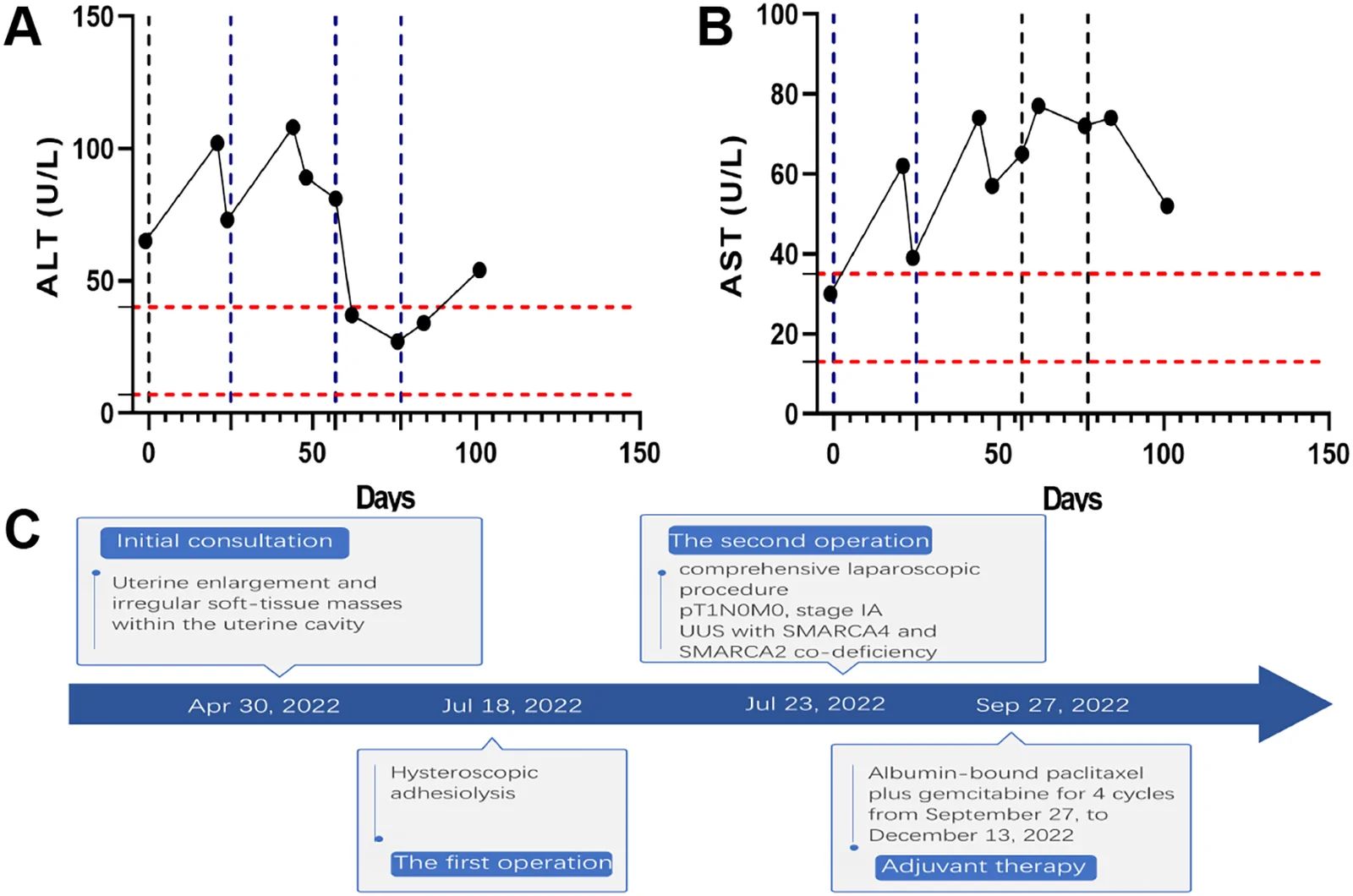
Laboratory findings and diagnostic and treatment timeline. Changes in serum ALT **(A)** and AST **(B)** levels during adjuvant chemotherapy. The y-axis represents serum ALT or AST concentration, whereas the x-axis represents time elapsed since the initiation of chemotherapy, with Day 0 defined as the first day of the initial chemotherapy cycle. Two red horizontal dashed lines indicate the upper and lower limits of the reference (normal) range. Four blue vertical dashed lines indicate the start of each chemotherapy cycle. Diagnostic and treatment Timeline **(C)**.

Regular follow-up assessments have been performed. Prior to July 2024, patients underwent scheduled follow-up examinations every three months, including abdominal CT or MRI, complete blood count, serum biochemical profiling, and tumor marker assessment. From July 2024 onward, the follow-up interval was extended to every six months. Chest CT was conducted every three months until June 2023 and subsequently transitioned to biannual imaging starting in July 2023. ALT and AST levels were frequently elevated during follow-up; however, elevations typically remained below threefold the upper limit of normal and subsequently declined following hepatoprotective therapy. The patient has remained free of disease recurrence as of the most recent follow-up on March 21, 2026 (**Figures 1E–H**). At that time, the patient had an Eastern Cooperative Oncology Group (ECOG) performance status of 0 and had achieved a DFS of 32 months.

## 3 Discussion

In this case report, we describe a patient with stage IA UUS harboring *SMARCA4* and *SMARCA2* co-deficiency. *SMARCA4*-deficient undifferentiated/dedifferentiated endometrial carcinoma exhibited marked immunohistochemical heterogeneity, whereas *SMARCA4*-deficient uterine sarcoma demonstrated uniformly consistent immunophenotypic profiles: all *SMARCA4*-deficient uterine sarcoma cases showed concurrent loss of BRG1 and BRM expression, with preserved expression of other SWI/SNF complex components; absent claudin-4 expression and SOX2 expression; and no evidence of mismatch repair deficiency or mutant-type p53 accumulation (14). In this case, immunohistochemical analysis demonstrated negative expression of pan-cytokeratin (CK-pan) and CK7, as well as estrogen receptor (ER) and progesterone receptor (PR), effectively excluding dedifferentiated endometrial carcinoma. Absence of desmin and smooth muscle actin (SMA) expression ruled out leiomyosarcoma. Negative CD10 and cyclin D1 immunoreactivity excluded high-grade endometrial stromal sarcoma. Notably, *SMARCA4* (BRG1) and *SMARCA2* (BRM) proteins were completely lost, whereas *SMARCB1* (INI-1) expression was retained; vimentin showed diffuse strong positivity. Microsatellite analysis revealed genomic stability at microsatellite loci. No evidence of extragenital malignancy was identified on clinical or radiological evaluation.

Based on these histopathological and immunophenotypic features, the diagnosis was established as primary *SMARCA4/2*-deficient UUS. For apparently early-stage UUS, the standard surgical approach is total hysterectomy without morcellation, combined with bilateral sapling-oophorectomy (5). Most patients rapidly develop distant metastases despite undergoing surgical resection (15). Adjuvant chemotherapy may provide clinical benefit for patients with HG-ESS (16). However, the prognosis of UUS remains extremely poor despite adjuvant chemotherapy, as demonstrated in a multi-institutional retrospective study conducted by the Japanese Clinical Oncology Group (16). Concomitant loss of *SMARCA4/2* occurs in a subset of cancers associated with very poor outcomes (13).

Inactivating mutations in the core *SMARCA* genes, *SMARCA2* and *SMARCA4*, result in loss of nuclear expression of their respective proteins. Consequently, these genetic alterations define a distinct class of malignancies characterized by *SMARCA* deficiency (9). *SMARCA4* is a tumor suppressor gene that is aberrantly altered in approximately 5% to 7% of human malignancies (17). *SMARCA4*-deficient UUS is a highly aggressive, molecularly defined malignancy driven by biallelic inactivation of *SMARCA4* (11). It also exhibits distinct clinicopathologic and molecular characteristics that distinguish it from undifferentiated endometrial carcinoma (18). In ESTs, genes involved in chromosomal rearrangements function as transcriptional regulators either directly through the formation of zinc finger motifs or indirectly through epigenetic mechanisms (19). SWI/SNF chromatin remodeling complexes interact with histones and sequence-specific transcription factors to dynamically regulate chromatin architecture and thereby control gene expression (20). *SMARCA4*, which encodes the BRG1 ATPase subunit, is a core component of the SWI/SNF chromatin remodeling complex and plays essential roles in transcriptional regulation and maintenance of genomic stability (21). BRG1 primarily exerts a positive regulatory effect on chromatin accessibility, RNA polymerase II (RNAPII) recruitment, and nascent RNA synthesis by modulating nucleosome positioning and promoting transcription factor binding to their cognate genomic sites (22). *SMARCA4* also regulates inducible BRD4-mediated genomic redistribution to coordinate intrinsic immunity and cellular plasticity during epithelial injury and repair (23).

Although our patient was diagnosed with stage IA disease following surgery, four cycles of adjuvant albumin-bound paclitaxel plus gemcitabine were administered. As recommended by the ESMO–EURACAN Clinical Practice Guidelines, surgery remains the standard treatment for all patients with localized adult-type soft tissue sarcoma (24). Surgical management constitutes the cornerstone of treatment for uterine sarcoma, and the primary therapeutic objective is complete, intact resection of the tumor, without intraoperative tumor fragmentation or dissemination (25). However, the present patient underwent hysteroscopic desmolases on July 18, 2022, followed by a comprehensive laparoscopic procedure under general anesthesia on July 23, 2022. Postoperative pathological evaluation confirmed stage IA disease, with vascular tumor emboli identified. Established prognostic factors associated with recurrence and survival in surgically staged uterine carcinosarcoma include depth of myometrial invasion, lymphovascular space invasion (LVSI), adnexal or serosal involvement, positive peritoneal cytology, and lymph node metastases (26). Notably, LVSI has been significantly associated with an increased risk of disease recurrence in patients with uterine sarcoma (27). Furthermore, a retrospective analysis of patients with localized HG-ESS and UUS (stages I–III) demonstrated that absence of vascular invasion was independently associated with improved DFS (28).

Therefore, in this patient, non-en-bloc resection, high-risk pathological subtype, and the presence of a postoperative vascular tumor thrombus may collectively represent potential risk factors for disease recurrence. To mitigate this risk, the patient received adjuvant chemotherapy following a multidisciplinary team discussion and with informed consent obtained from the patient’s family. Indeed, according to the National Comprehensive Cancer Network (NCCN) Clinical Practice Guidelines in Oncology (Version 2.2026: Uterine Sarcoma), postoperative observation remains the standard recommendation for stage I UUS. Although -deficient uterine sarcomas are frequently associated with highly aggressive clinical behavior, the NCCN guidelines (Version 2.2026) do not endorse a specific adjuvant therapy regimen for stage I disease. However, per the European Society for Medical Oncology (ESMO)–European Reference Network for Rare Adult Solid Cancers (EURACAN) Clinical Practice Guidelines, adjuvant chemotherapy may be considered for select high-risk patients (e.g., those with high-grade disease) (24). Adjuvant chemotherapy, with or without radiotherapy, may be considered (5). In the **Supplementary Table S1**, among previously reported cases of *SMARCA4*-deficient and *SMARCA4/2*–deficient UUSs, OS ranged from 15 days to 14 months. The present case exhibits a longer OS than previously documented. Most reported patients were diagnosed at stage III or IV, consistent with the advanced disease pattern described in the literature (5, 25), whereas this patient was diagnosed at pathological stage IA. Notably, all three previously reported cases of stage IB *SMARCA4*-deficient UUSs received adjuvant chemotherapy (**Supplementary Table S1**).

Current therapeutic advances in clinical research on *SMARCA4/2*-deficient UUS include the following: (I) Immunotherapy: In a comparison of two cases of *SMARCA4*-deficient UUSs with other ESS subtypes, *SMARCA4*-deficient UUSs demonstrated a highly immunogenic tumor microenvironment characterized by prominent lymphocytic infiltration and dynamic intercellular interactions (29). These findings highlight the potential therapeutic value of immunotherapy for patients with *SMARCA4*-deficient UUSs (29). (II) Poly (ADP-ribose) polymerase (PARP) inhibitors: Loss of *SMARCA4* induces RNAPII stalling, leading to R-loop accumulation that sequesters breast cancer type 1 eusceptibility protein (BRCA1) at transcription complexes, thereby contributing to the heightened sensitivity of *SMARCA4*-deficient ovarian cancers to inhibitors targeting PARP and RNAPII elongation (30).

Recent advances in preclinical research on the therapeutic management of *SMARCA4/2*-deficient cancers are summarized below. During tumorigenesis, loss of function of either *SMARCA4* or *SMARCA2* is frequently observed; however, concurrent loss of both genes has been reported to be synthetically lethal and is therefore considered unexpected (12). (I) Histone deacetylase inhibitors: The contribution of *SMARCA4/2* to Ca^2+^-dependent apoptosis may represent a therapeutic vulnerability that could be exploited to enhance the efficacy of chemotherapy in *SMARCA4/2*-deficient cancers (31). Histone deacetylase inhibitor–mediated reactivation of *SMARCA2* restores IP3R3 expression and potentiates cisplatin sensitivity in *SMARCA4/2*-deficient cancer cells (31). (II) Oxidative phosphorylation (OXPHOS) inhibitors: *SMARCA4/2* loss suppresses glucose transporter 1 expression, leading to decreased glucose uptake and glycolysis, while concurrently increasing cellular dependence on OXPHOS. *SMARCA4/2*-deficient cells and tumors exhibit heightened sensitivity to OXPHOS inhibitors (13). (III) Enhancer of zeste homolog 1/2 (EZH1/2) inhibitors: The EZH1/2 inhibitor HM97662 selectively suppresses growth of *SMARCA4/2*-deficient cells by inhibiting stemness and proliferation, revealing a therapeutic vulnerability in *SMARCA4/2*-deficient tumors (32). (IV) Inhibition of myeloid cell leukemia-1 (MCL1): MCL1, an anti-apoptotic member of the B-cell lymphoma 2 (Bcl-2) protein family, represents a synthetic lethal target in *SMARCA4/2*-deficient cancers and holds therapeutic potential (33). (V) Alanine supplementation: *SMARCA4/2*-deficient cells rely on the upregulated amino acid transporter SLC38A2 to enhance glutamine uptake and maintain mitochondrial OXPHOS. Notably, alanine, imported specifically via SLC38A2, competitively inhibits glutamine transport and selectively induces cell death in *SMARCA4/2*-deficient cancer cells. These findings suggest that alanine supplementation may potentiate current therapeutic strategies for aggressive cancers harboring loss-of-function alterations in *SMARCA4/2* (13).

It must be acknowledged that this case report is subject to several limitations. (I) In this case report, loss of *SMARCA4/2* expression was confirmed by immunohistochemistry alone; Owing to the patient’s refusal of the proposed tumor and germline genetic sequencing. next-generation sequencing was not performed. Consequently, no pathogenic mutations were identified, and no additional molecular alterations were assessed. (II) As a case report, this study provides a low level of evidence according to the hierarchy of evidence in evidence-based medicine.

In conclusion, this case suggests that patients with UUS harboring *SMARCA4* and *SMARCA2* co-deficiency may derive a survival benefit from early diagnosis and surgical resection. Nevertheless, additional robust clinical evidence is required to substantiate the survival benefit associated with adjuvant chemotherapy. However, due to the extreme rarity of UUS with *SMARCA4* and *SMARCA2* co-deficiency, conducting a high-level phase III randomized controlled trial to validate this observation remains impractical.

## 4 Patient perspective

When I first heard I had gynecologic cancer, I totally lost it—super anxious and emotionally wiped out. After surgery and chemo, my liver enzymes shot up for a bit, just a side effect from the chemo, nothing to worry about. All things considered, I bounced back pretty well, and slowly got back into the swing of things: hanging out with family, enjoying retirement, and doing the stuff that really matters to me.

## Data Availability

The raw data supporting the conclusions of this article will be made available by the authors, without undue reservation.

## Glossary

AJCC: American Joint Committee on Cancer
ALT: alanine aminotransferase
ARID1A: AT-rich interactive domain-containing protein 1A
ARID1B: AT-rich interactive domain-containing protein 1B
AST: aspartate aminotransferase
Bcl-2: B-cell lymphoma 2
BRCA1: breast cancer type 1 eusceptibility protein
CTCAE: Common Terminology Criteria for Adverse Events
CT: computed tomography
DFS: disease-free survival
ECOG: Eastern Cooperative Oncology Group
ER: estrogen receptor
ESMO: European Society for Medical Oncology
ESN: endometrial stromal nodule
EST: endometrial stromal tumor
EURACAN: European Reference Network for Rare Adult Solid Cancers
EZH1/2: enhancer of zeste homolog 1/2
H&E: hematoxylin and eosin
HG-ESS: high-grade endometrial stromal sarcoma
MCL1: myeloid cell leukemia-1
MRI: magnetic resonance imaging
NCCN: National Comprehensive Cancer Network
OS: overall survival
OXPHOS: oxidative phosphorylation
PARP: poly (ADP-ribose) polymerase
PR: progesterone receptor
RNAPII: RNA polymerase II
SMARCA2: SWI/SNF-related, matrix-associated, actin-dependent regulator of chromatin, subfamily A, member 2
SMARCA4: SWI/SNF-related, matrix-associated, actin-dependent regulator of chromatin, subfamily A, member 4
SWI/SNF: SWItch/sucrose non-fermentable
UICC: Union for International Cancer Control
UUS: undifferentiated uterine sarcoma
WHO: World Health Organization.
