# Supplementary material for: Stage IA undifferentiated uterine sarcoma with SMARCA4 and SMARCA2 co-deficiency: a case report
Source: Front Oncol. 2026 Oct 2;16:1939651. doi: 10.3389/fonc.2026.1939651 (PMC13671925; doi:10.3389/fonc.2026.1939651)
Supplement: Supplementary file 1 [file Table1.docx]

Table S1. Cases with SMARCA4-deficient or SMARCA4/2-deficient UUSs documented in the literature

|  | Age | Stage | Deficiency | Surgery | Noninvasive therapeutic interventions | Outcomes | Authors | References |
| --- | --- | --- | --- | --- | --- | --- | --- | --- |
| 1 | 38 | IVB | SMARCA4 | No surgical intervention | PRaG5.0 combined with chemotherapy and sequential chemoimmunotherapy | PFS > 12 months | Li Z, et al | (1) |
| 2 | 61 | IB | SMARCA4 | A single-port laparoscopic total hysterectomy, bilateral adnexectomy and pelvic lymph node dissection | 3 cycles of postoperative platinum-based chemotherapy combined with docetaxel and epirubicin | No records available | Wang Y, et al | (2) |
| 3 | 40+ | IB1 | SMARCA4 | Radical hysterectomy | Adjuvant concurrent chemoradiotherapy with cisplatin | OS > 1 year | Yokoe T, et al | (3) |
| 4 | 27 | pT3bNxMx | SMARCA4 | Hysterectomy and bilateral salpingooophorectomy | Postoperative chemotherapy consisting of 4 cycles of gemcitabine and docetaxel; first-line therapy consisting of 3 cycles of epirubicin and ifosfamide | OS > 6 months | Gao J, et al | (4) |
| 5 | 52 | IIIC | SMARCA4 | Hysteroscopic-guided polypectomy followed by hysterectomy with bilateral salpingo-oophorectomy and pelvic lymph node dissection | None | OS = 15 days, death | Nakra T, et al | (5) |
| 6 | 24 | IB | SMARCA4 | A total abdominal hysterectomy, bilateral salpingo-oophorectomy, pelvic and paraaortic lymphadenectomy, and partial omentectomy | Adjuvant chemotherapy with paclitaxel and carboplatin | DFS = 13 months | Suzui R, et al | (6) |
| 7 | 46 | IIIC | SMARCA4 | No surgical intervention | First-line chemotherapy with gemcitabine and docetaxel | PFS = 3 months | Kord A, et al | (7) |
| 8 | 51 | IIIC | SMARCA4 | A total abdominal hysterectomy, bilateral salpingo-oophorectomy, and pelvic and low para-aortic lymph node dissection | Adjuvant chemotherapy consisting of 6 cycles of doxorubicin and ifosfamide; First-line therapy consisting of 1 cycle of gemcitabine and docetaxel; Low-dose radiation therapy (8 Gy, once); Seond-line therapy with pazopanib; Third-line therapy with eribulin; Whole-liver irradiation (8 Gy in a single fraction); | DFS = 6 months, OS = 12 months | Kurokawa M, et al | (8) |
| 9 | 23 | IVA | SMARCA4/2 | Radical surgery, including total abdominal hysterectomy and bilateral salpingo-oophorectomy | Systemic chemotherapy with doxorubicin monotherapy, followed by combination therapy with gemcitabine and docetaxel | OS = 6 months, death | Yoshida H, et al | (9) |
| 10 | 28 | IIIC | SMARCA4/2 | A total abdominal hysterectomy, bilateral salpingo-oophorectomy, pelvic and paraaortic lymphadenectomy, and partial omentectomy | Adjuvant chemotherapy with paclitaxel and carboplatin; first-line therapy with pembrolizumaband lenvatinib for 5 months | DFS = 9 months, PFS1 > 5 months | Suzui R, et al | (6) |

References

1.	Li Z, Pang X, Wang Z, Wen J, Zhao Y, Ma D, et al. PRaG5.0 combined with chemotherapy and sequential chemoimmunotherapy for massive SMARCA4-deficient undifferentiated tumor of the cervix: case report. Front Immunol. 2026;17:1826847.

2.	Wang Y, Sun Y, Jiang Y, Chen X, Dai H. SMARCA4-deficient undifferentiated uterine sarcoma: A case report and literature review. Medicine (Baltimore). 2025;104(33):e43828.

3.	Yokoe T, Oku K, Noda Y, Kita M. Treatment for SMARCA4-deficient carcinoma of uterine cervix. BMJ Case Rep. 2025;18(1):1-4.

4.	Gao J, Fan R, Chen D, Hou J, Chen H, Lu M. Pathological characteristics and immune microenvironment of SMARCA4-deficient undifferentiated uterine sarcoma. Diagn Pathol. 2023;18(1):62:1-6.

5.	Nakra T, Kakkar A, Mathur SR, Jain D, Kumar S. SMARCA4-deficient Undifferentiated Uterine Sarcoma: Clinicopathological Features of an Emerging Entity. Int J Surg Pathol. 2023;31(1):104–9.

6.	Suzui R, Taki M, Kitamura S, Sunada M, Yamanoi K, Murakami R, et al. SMARCA4-deficient uterine tumors in young women: response to immune checkpoint inhibitors. Int Cancer Conf J. 2024;13(4):515–9.

7.	Kord A, Eppurath A, Drammeh H, Elbaz Younes I, Xie KL. SMARCA4-deficient uterine sarcoma: A case report and a concise review. Case Rep Womens Health. 2020;27:e00231.

8.	Kurokawa M, Shimizuguchi T, Ito K, Takao M, Motoi T, Taguchi A, et al. Notable Response of SMARCA4-Deficient Undifferentiated Uterine Sarcoma to Palliative Radiation Therapy. Adv Radiat Oncol. 2021;6(5):100728.

9.	Yoshida H, Shiraishi K, Yamanaka T, Ishikawa M. Illustrating the "Quiet Genome" Concept of SMARCA4 -Deficient Uterine Sarcoma Via Whole-Exome Sequencing: A Highly Aggressive Case in a Young Woman. Int J Gynecol Pathol. 2026,00:1-6.
